# Intronic Polymorphisms in the *CDKN2B-AS1* Gene Are Strongly Associated with the Risk of Myocardial Infarction and Coronary Artery Disease in the Saudi Population

**DOI:** 10.3390/ijms17030395

**Published:** 2016-03-17

**Authors:** Sayed AbdulAzeez, Awatif N. Al-Nafie, Abdullah Al-Shehri, J. Francis Borgio, Ekaterina V. Baranova, Mohammed S. Al-Madan, Rudaynah A. Al-Ali, Fahad Al-Muhanna, Abdullah Al-Ali, Mohammed Al-Mansori, Mohammed Fakhry Ibrahim, Folkert W. Asselbergs, Brendan Keating, Bobby P. C. Koeleman, Amein K. Al-Ali

**Affiliations:** 1 Department of Genetic Research, Institute for Research and Medical Consultation (IRMC), University of Dammam, P.O. Box 1982, 31441 Dammam, Saudi Arabia; borgiomicro@gmail.com; 2 Department of Pathology, King Fahd Hospital of the University, University of Dammam, 34445 Al-Khobar, Saudi Arabia; analnafie@uod.edu.sa; 3 Department of Internal Medicine, King Fahd Hospital of the University, University of Dammam, 34445 Al-Khobar, Saudi Arabia; ashehri60@yahoo.com (A.A.-S.); rudaynahalali@hotmail.com (R.A.A.-A.); fmuhanna@uod.edu.sa (F.A.-M.); mmansori@uod.edu.sa (M.A.-M.); mfibrahim@uod.edu.sa (M.F.I.); 4 Department of Pharmaceutical Sciences, Division of Pharmacoepidemiology and Clinical Pharmacology, Utrecht University, 3508 Utrecht, The Netherlands; E.V.Baranova@uu.nl; 5 Department of Pediatrics, King Fahd Hospital of the University, University of Dammam, 34445 Al-Khobar, Saudi Arabia; mmadan@uod.edu.sa; 6 Department of Cardiology, King Fahd Hospital, Al-Hafof, 36441 Al-Ahssa, Saudi Arabia; qardoc@hotmail.com; 7 Division of Heart & Lungs, Department of Cardiology, University Medical Center Utrecht, 3508 GA Utrecht, The Netherlands; F.W.Asselbergs@umcutrecht.nl; 8 Penn Transplant Institute, Hospital of the University of Pennsylvania, Philadelphia, 19104 PA, USA; bkeating@mail.med.upenn.edu; 9 Department of Medical Genetics, University Medical Center Utrecht, 3508 Utrecht, The Netherlands; B.P.C.Koeleman@umcutrecht.nl; 10 Institute for Research and Medical Consultation (IRMC), University of Dammam, 31441 Dammam, Saudi Arabia; aalali@uod.edu.sa

**Keywords:** coronary artery disease, myocardial infarction, single nucleotide polymorphisms, *CDKN2B-AS1* gene, Saudi Arabia

## Abstract

Recent genome-wide association studies identified single nucleotide polymorphisms (SNPs) on the chromosome 9p21.3 conferring the risk for CAD (coronary artery disease) in individuals of Caucasian ancestry. We performed a genetic association study to investigate the effect of 12 candidate SNPs within 9p21.3 locus on the risk of CAD in the Saudi population of the Eastern Province of Saudi Arabia. A total of 250 Saudi CAD patients who had experienced an myocardial infarction (MI) and 252 Saudi age-matched healthy controls were genotyped using TaqMan assay. Controls with evidenced lack of CAD provided 90% of statistical power at the type I error rate of 0.05. Five percent of the results were rechecked for quality control using Sanger sequencing, the results of which concurred with the TaqMan genotyping results. Association analysis of 12 SNPs indicated a significant difference in the genotype distribution for four SNPs between cases and controls (rs564398 *p* = 0.0315, χ^2^ = 4.6, odds ratio (OD) = 1.5; rs4977574 *p* = 0.0336, χ^2^ = 4.5, OD = 1.4; rs2891168 *p* = 1.85 × 10 − 10, χ^2^ = 40.6, OD = 2.1 and rs1333042 *p* = 5.14 × 10 − 9, χ^2^ = 34.1, OD = 2.2). The study identified three protective haplotypes (TAAG *p* = 1.00 × 10 − 4; AGTA *p* = 0.022 and GGGCC *p* = 0.0175) and a risk haplotype (TGGA *p* = 2.86 × 10 − 10) for the development of CAD. This study is in line with others that indicated that the SNPs located in the intronic region of the CDKN2B-AS1 gene are associated with CAD.

## 1. Introduction

Coronary artery disease (CAD) is a major cause of morbidity and mortality worldwide and represents a tremendous social and economic burden on society [1]. In Saudi Arabia, the prevalence of CAD is 5.5% in comparison to European Caucasians at a prevalence of 6.4% [2]. Although several independent risk factors for CAD (including smoking, hypercholesterolemia, hypertension, obesity, and diabetes) have been identified, the genetic basis for CAD remains relatively unknown. Recently, genome-wide association studies (GWAS) have reported the increased susceptibility to CAD in carriers of certain single nucleotide polymorphisms (SNPs) within chromosome 9p21.3 locus [3,4,5,6,7]. This locus codes for an anti-sense RNA (*CDKN2B-AS1* or *ANRIL*), which is located nearby the *CDKN2A-CDKN2B* gene cluster [8,9,10]. The *CDKN2A* and *CDKN2B* genes were shown to be significantly associated with an increased risk of CAD (21%) [3,4,11]. Several studies have identified a significant association of specific SNPs in the 9p21.3 locus with CAD [9,10,11,12,13,14,15,16,17,18,19,20,21,22,23,24,25,26,27,28]. However, the majority of these studies were primarily conducted on Caucasian, African American, and Chinese populations. Studies conducted on the population of the Eastern Province of Saudi Arabia have reported that 59.2% of sudden death incidences are attributable to CAD [29]. We carried out a case-control study to investigate the association of 12 risk variants at 9p21.3 locus with myocardial infarction (MI) in a Saudi Arabian population for the first time.

## 2. Results

After obtaining written informed consent from all patients and volunteers, blood samples and clinical data were collected. The clinical characteristics of the study population are presented in Table 1. The study included 250 Saudi CAD patients who had experienced an MI and 252 age matched healthy controls with no history of CAD. These numbers have provided 90% of statistical power at the type I error rate of 0.05 with the maximum odd ratio value of 2. The observed genotype frequencies of the tested polymorphisms in this study obeyed the Hardy–Weinberg equilibrium (Table 2). Initially, the statistical analyses of the association of all the SNPs with CAD patients, who had a secondary condition, either hypertension, type 2 diabetes (T2D), or obesity, were evaluated separately. However, as there were statistical differences between the controls and the patient sub-groups, the CAD patients, regardless of their secondary condition, were pooled prior to data analysis.

**Figure 1:**
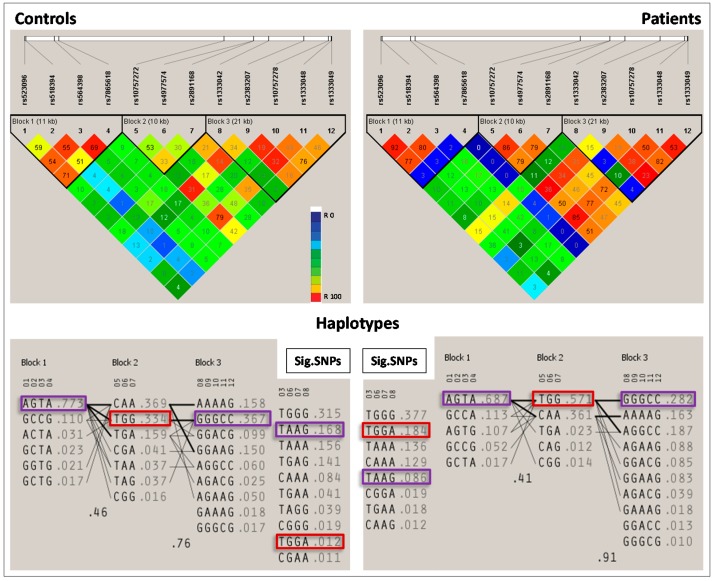
**Upper half**: Haploview LD (linkage disequilibrium) plot of the 12 SNPs in 9p21 locus. The pairwise correlation between the single nucleotide polymorphisms (SNPs) were measured as *r*^2^ and shown (×100) in each diamond. **Lower half**: Haplotypes of patient group and controls. Protective haplotypes are in purple boxes and risk haplotypes are in red boxes. Sig. SNPs: Block and haplotypes of significant SNPs.

**Figure 2:**
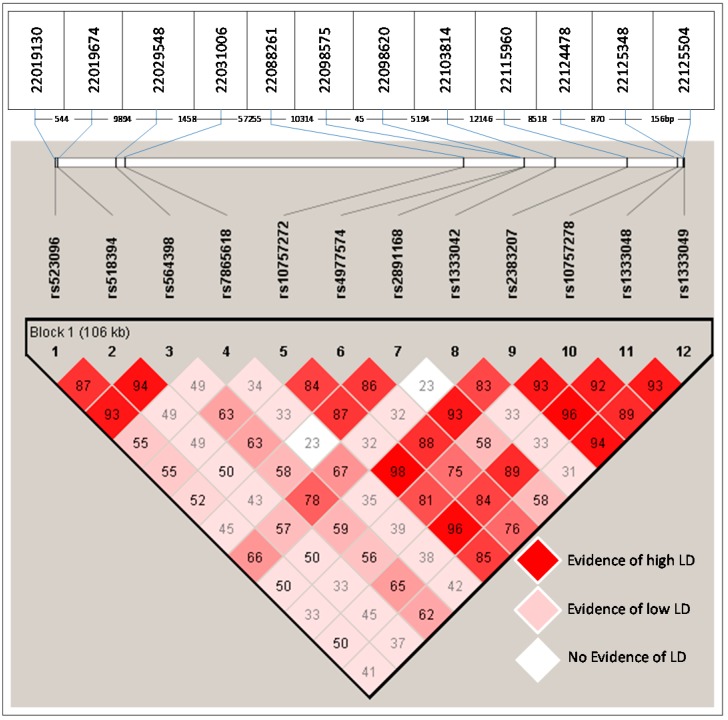
The chromosomal positions of the 12 SNPs analyzed in 9p21 locus and their LD. **Upper table**: Indicating the coordinates according to the reference sequence NT_008413.18. **Middle row**: indicates the distance (bp) between two adjacent SNPs. **Lower half**: Indicating the *D’* value in each diamond among 12 SNPs in 9p21.

The genetic association analysis of 12 SNPs indicated a significant difference in the genotypic distribution of four SNPs between cases and controls (rs564398 *p* = 0.0315, χ^2^ = 4.6, OD = 1.5; rs4977574 *p* = 0.0336, χ^2^ = 4.5, OD = 1.4; rs2891168 *p* < 0.0001, χ^2^ = 40.6, OD = 2.1 and rs1333042 *p* < 0.0001, χ^2^ = 34.1, OD = 2.2) (Table 2; Figure 1). The test identified a significant association between GG genotype of rs2891168 (OR: 2.7919; 95%CI (Confidence interval): 1.8201–4.2824; χ^2^: 23.5464 and *p* < 0.0001) and CAD using a common genetic model, namely the recessive, dominant, and additive model. Permutation association test of single markers conferred the most significant risk effect of rs289116G on CAD (permutation *p-*value < 0.0001). An extended RR of risk alleles revealed that the rs2891168G allele has the most significant association (*p* < 0.0001; RR: 1.4593 CI at 95% 1.2848–1.6575). An LD (linkage disequilibrium) test (*D prime* (*D’*) = 0.86) showed that the alleles of rs2891168G and rs4977574G are strongly linked with each other among the significant SNPs in the Saudi Arabian population (Figure 2).

LD plot was constructed based on the pairwise correlation between the 12 SNPs on the 9p21 locus, which has three blocks (blocks 1, 2, and 3) of linked variants (Figure 1). Furthermore, a single group of the four significant SNPs (Sig. SNPs) was made as one block and analyzed for its significant association with the CAD (Figure 1). Haplotype analysis of the significant SNPs, rs564398, rs4977574, rs2891168, and rs1333042 indicated that the haplotype TGGA is a risk factor for CAD. The haplotypes TAAG (rs564398, rs4977574, rs2891168, and rs1333042); AGTA (rs523096, rs518394, rs564398, and rs7865618); and GGGCC (rs1333042, rs2383207, rs10757278, rs1333048, and rs1333049) are CAD protective (*p* < 0.0001, 0.022, and 0.0175, respectively) (Figure 1 and Table 3). The protective haplotypes in Block 1 (AGTA *p* = 0.022) and in Block 3 (GGGCC *p* = 0.0175) with more than 30% frequency were observed in controls (Figure 1 and Table 3). Furthermore, the most significant risk haplotype TGG (rs10757272:rs4977574:rs2891168 *p* < 0.0001) of Block 2 had a frequency of 56.8% in patients and 34.5% in controls.

To evaluate whether the SNPs identified to be associated with CAD (rs564398, rs4977574, rs2891168, and rs1333042) enhance the predictive value of known conventional risk factors such as gender, age, and body mass index (BMI), two multivariate models were built. The first model (the clinical only model) consisted of known CAD risk factors collected in this study: gender, age, and BMI. For the second model (clinical + genetic model), the four CAD-associated SNPs (rs564398, rs4977574, rs2891168, and rs1333042) were entered into the model assuming an additive model of inheritance, in addition to those included in the clinical-only model (Table 4). SNPs rs4977574 and rs564398 were not significantly associated with CAD (*p* > 0.05) after adjusting for other variables in the model and therefore were removed from the clinical + genetic model. The final clinical + genetic model included age, gender, BMI, rs1333042, and rs2891168. As shown in Table 4, both models were significantly predictive of CAD, with area under curve (AUC) of 0.79 (95% CI: 0.73–0.84) for clinical only model (*p* < 10^−10^), and AUC of 0.87 (95% CI: 0.82–0.90) for clinical + genetic model. Most importantly, two CAD-associated SNPs, rs1333042, and rs2891168, improved the predictive power for CAD over the model composing of only conventional known risk factors, with an improvement in AUC of 0.08 (95% CI: 0.04–0.12, *p* = 0.000236).

## 3. Discussion

During the last five decades, Saudi Arabia has undergone tremendous socio-economic changes, which have resulted in an increase in the prevalence of common diseases, such as diabetes, cardiovascular diseases, and obesity [1,2]. However, the high prevalence of cardiovascular diseases in the Kingdom cannot be solely attributed to these socio-economic changes. It is believed that genetics and ion channels are contributory factors in the increased prevalence rate of these diseases [30,31]. Recent genome-wide association studies have indicated that there is an association between the increased susceptibility to CAD and specific single nucleotide polymorphisms (SNPs) within the genome [3,4,5,6,7] which play a role in conjunction with other known traditional CAD risk factors. One such locus has been identified within chromosome 9p21.3 which codes for an anti-sense RNA (*CDKN2B-AS1* or *ANRIL*) and is located near the *CDKN2A-CDKN2B* gene cluster [3,7,8,9,10]. The variants within this locus were shown to be significantly associated with an increased risk of CAD and diabetes in populations of European origin and some other populations [3,4,11]. It is of great importance that these studies are replicated, especially in populations that have distinctive genetic backgrounds, such as that in the Eastern Province of Saudi Arabia, in order to understand the pathophysiology of CAD. This is the first study undertaken on an Arab population to replicate previous studies conducted on the association of these variants with CAD in other populations [9,10,11,12,13,14,15,16,17,18,19,20,21,22,23,24,25,26,27]. Type 2 diabetes mellitus, dyslipidemia, and hypertension were considered separately for the significance analysis. These three parameters did not show any significant association during the non-random association of 12 alleles.

Four of the 12 SNPs tested in the present study were significantly associated with CAD (*p* < 0.05). One of the four SNPs (rs564398) has been shown to be moderately associated with T2D in Israeli population [32]. The high OR of the risk alleles harboring these four SNPs may be due to the high percentage of consanguinity in the Eastern Province of Saudi Arabia, which increases the possibility of inheriting a recessive allele [33,34,35,36,37].

Haplotype analysis of the rs564398, rs4977574, rs2891168, and rs1333042 indicated that the haplotype TGGA is a risk factor for CAD, while the haplotypes TAAG (rs564398, rs4977574, rs2891168, and rs1333042); AGTA (rs523096, rs518394, rs564398, and rs7865618); and GGGCC (rs1333042, rs2383207, rs10757278, rs1333048, and rs1333049) are CAD protective (*p* < 0.0001, 0.022, and 0.0175, respectively). These haplotypes are reported here for the first time in an Arab population. However, the SNPs of haplotypes TAAG and TGGA (rs564398, rs4977574, rs2891168, and rs1333042) have been found to be associated with CAD in other populations, including European Hispanic, Chinese, white American, and Turkish populations [9,10,11,12,13,14,15,16,17,18,19,20,21,22,23,24,25,26,27].

In comparing two multivariate models built with and without CAD-associated SNPs, we found these risk SNPs adding predictive value of CAD on top of known risk factors such as age, gender, and BMI. Due to limited collection of clinical variables, traditional risk factors commonly included in the CAD risk prediction model, such as systolic blood pressure and blood lipid levels HDL (High-density lipoprotein), LDL (Low-density lipoprotein), and TC (total cholesterol), were not evaluated in the model building process. Further studies that collect these clinical risk factors in order to evaluate the improvement in prediction value from risk SNPs are needed.

## 4. Materials and Methods

### 4.1. Study Population

The study, which was conducted from 2012–2014, included 250 Saudi CAD patients who had experienced an MI, (STEMI and NSTEMI) as diagnosed by the medical history such as, signs and symptoms (like fatigue), chest discomfort (with physical examination), in addition to electrocardiogram and high troponin level. The patients were selected randomly from major hospitals in Al-Ahssa Qatif and Al-Khobar in the Eastern Province of Saudi Arabia. Two-hundred and fifty-two age-matched healthy volunteers attending the blood banks of the same hospitals were included in the study as controls. The majority of patients attending these hospitals either live in these areas or originate from these areas, with very little population admixture due to the high rate of consanguinity. This has been confirmed by our recent research results [33,34]. Stringent inclusion criteria for both cases and controls were followed as a result of the high incident rate of CAD (11.7%) in this age group (>40 years) in the Saudi population [38,39,40,41,42]. CAD diagnosis was based on electrocardiogram, echocardiography, blood tests and coronary angiography and cardiac catheterization. Exclusion criteria for the control group included a present or past or family history of CAD. Peripheral blood samples (5 mL) were collected for genetic analysis. SNPs selected for inclusion in the study have been previously reported to be strongly associated with CAD [9,10,11,12,13,14,15,16,17,18,19,20,21,22,23,24,25,26,27,28].

This study was approved by the Institutional Review Board and Committee for Biological and Medical Ethics, University of Dammam. Written informed consent was obtained from all study participants.

### 4.2. TaqMan^®^ Single Nucleotide Polymorphisms (SNPs) Genotyping

The genomic DNA was extracted from peripheral blood samples of patients and controls using a standard extraction kit (Wizard Genomic DNA purification kit, Promega, Madison, WI, USA). The Rotor-Gene Q (Qiagen, Hilden, Germany) real time polymerase chain reaction (PCR) system was used to perform SNP genotyping using TaqMan^®^ assay (Applied Biosystems, Foster City, CA, USA). Genotyping of 12 SNPs (rs1333042, rs2891168, rs4977574, rs10757278, rs2383207, rs523096, rs518394, rs564398, rs7865618, rs1333049, rs10757272, and rs1333048) was carried out separately in a 15 μL volume containing 20 ng of genomic DNA, 6.25 μL of 2× TaqMan master mix, 1.25 μL of 20× assay working stock, and nuclease free water to 15 μL. An allele calling report was generated with Rotor-Gene Q Series Software 2.0.2 (Build 4) (Corbett Life Science, Qiagen).

### 4.3. Quality for SNP Genotyping—Sanger Sequencing

The initial TaqMan genotyping results were verified through Sanger sequencing on 5% of the samples. Oligonucleotide primers were designed for the PCR amplification product of ~150 bp up and downstream to the position of each SNP (primers are available on request) and were amplified separately. The PCR mixture contained 100 ng of DNA, top *Taq* buffer (10×) (5 μL), 25 mM MgCl_2_ (3 µL), 25 mM dNTP (0.4 µL), top *Taq* DNA polymerase 5 U/µL (0.2 µL), 10 µM forward and reverse (respective primers, accordingly) oligonucleotides (2 µL), and water (to 50 µL). The temperature profile was as follows: 5 min at 95 °C, 30 PCR cycles of 95 °C/30 s, 56 °C/30 s, and 72 °C/30 s, final extension of 7 min at 72 °C. The amplicons were purified using a standard PCR purification kit (QIAquick PCR Purification Kit, Qiagen). Cycle sequencing was performed in a BIO-RAD MyCycler™ (Bio-Rad, Hercules, CA, USA) with a total volume of 20 μL. Purified products (10 μL) were separated in a Genetic Analyzer 3500 DNA sequencer (Applied Biosystems). Data were analyzed using Sequencing Analysis Software V5.4 (Applied Biosystems). MAFFT version 7 was used for multiple sequence alignment. The quality for SNP genotyping through TaqMan^®^ assay was assured by direct DNA sequence analysis of 5% of the samples. Furthermore, the DNA samples for controls and cases were analyzed in the same batches.

### 4.4. Statistical Analysis

Statistical power analysis to ascertain association study was carried out to verify the adequate power of the selected sample size using the Sampsize online tool [42]. Clinical characteristics of the study participants were recorded upon inclusion. Differences in clinical characteristics between cases and controls were calculated by the two-sample t-test or the χ^2^ test if appropriate, odds ratios (OR) and relative risks (RR) for the association of risk alleles with CAD were calculated and 95% confidence intervals (CI) were constructed [43]. The allelic association of SNPs on cases and controls were assessed using the χ^2^ test. The Hardy-Weinberg equilibrium test was applied to confirm the independent segregation of the alleles. Non-random association of 12 alleles at 9p21 was tested by LD test. Haplotype blocks were constructed from the genotype data. To understand how genotypes of each SNP alleles are associated with CAD, we used a genetic model (Recessive, Dominant, and Additive model) in the association analysis. SNP haplotype analysis was extended to reveal the risk of SNP patterns on the development of CAD by treating each haplotype as a single variant and the other haplotypes as an alternative allele. Statistical significance was set at *p* ≤ 0.05. A permutation *p*-value for a single marker only was also calculated. All genetic analyses were performed using Haploview, version 4.2 [44].

Multivariate models to discriminate CAD cases and healthy controls were built using logistic regression. Demographic and patient characteristic variables (gender, age, and BMI) that were significantly associated with CAD were included in the model building (clinical only model). Individual SNPs that were found to be associated with CAD were then added to clinical model. The variables that were not significant after adjusting for other variables in the model were then removed, resulting in the final most parsimonious model including both demographic/clinical variables and SNPs (clinical + genetic model). The improvement in model performance of the clinical + genetic model compared to clinical only model was evaluated as outlined by Delong [45].

## 5. Conclusions

Four SNPs tested in the present study were significantly associated with CAD (*p* < 0.05). Haplotype analysis indicated that the haplotypes TGGA is a risk factor for CAD, while the haplotypes TAAG, AGTA, and GGGCC are CAD protective in the Saudi population of the Eastern Province. The study clearly replicates the findings of previous studies conducted on the association of these polymorphisms and CAD.

## Figures and Tables

**Table 1:** Clinical characteristics of coronary artery disease (CAD) cases and controls in a population from Eastern Province, Saudi Arabia.

Characteristics *	Cases (*n* = 250)	Controls (*n* = 252)
Males, *n* (%)	163 (65.2)	200 (79.4)
Females, *n* (%)	87 (34.8)	52 (20.6)
Age, (years) ^#^	52.3 ± 13.8	48.1 ± 7.9
BMI, (kg/m^2^)	33.6 ± 11.0	27.9 ± 4.0
Hypertension, *n* (%)	174 (69.6)	0 (0)
Type 2 diabetes *n*, (%)	146 (58.4)	1 (0.4)
Dyslipidemia	29 (11.6)	0 (0)
Unstable angina	4 (1.6)	0 (0)

* Data are shown as means ± standard deviations (SD) or percentages. ^#^ For cases age-at-diagnosis and for controls age-at-collection. Individuals with systolic blood pressure >140 mm Hg and diastolic blood pressure >90 mm Hg were defined as hypertension. BMI: Body mass index.

**Table 2:** Association of CAD with risk alleles of single nucleotide polymorphisms (SNPs) in 9p21.3 locus.

SNP ID	Assoc Allele	*p*-HW	ObsHET	PredHET	Odds Ratio (95% CI)	Case; Control Ratio	χ^2^	*p* Value
rs523096	G	0.2009	0.312	0.314	1.0369 (0.7466–1.4399)	99:401, 80:336	0.047	0.8287
rs518394	G	0.5137	0.316	0.314	1.0112 (0.7294–1.4018)	403:97, 341:83	0.005	0.9465
rs564398	C	0.1935	0.257	0.261	1.4917 (1.0345–2.1511)	89:411, 54:372	4.625	0.0315 *
rs7865618	G	0.1822	0.273	0.272	1.1249 (0.7906–1.6004)	85:415, 65:357	0.429	0.5127
rs10757272	T	0.2009	0.453	0.484	1.1612 (0.8882–1.5180)	301:197, 225:169	1.011	0.3146
rs4977574	G	0.1745	0.473	0.486	1.3515 (1.0462–1.7459)	308:192, 233:193	4.515	0.0336 *
rs2891168	G	0.0698	0.529	0.500	2.1908 (1.6920–2.8368)	301:197, 167:257	40.619	1.85 × 10^−10^ **
rs1333042	A	0.9668	0.721	0.485	2.2012 (1.6932–2.8616)	251:249, 133:293	34.137	5.14 × 10^−9^ **
rs2383207	A	0.0827	0.284	0.312	1.0005 (0.7165–1.3971)	96:400, 77:321	0	0.9976
rs10757278	G	0.3587	0.445	0.499	1.1438 (0.8824–1.4827)	249:251, 196:226	1.031	0.3099
rs1333048	C	0.1595	0.441	0.474	1.2095 (0.9225–1.5859)	317:183, 232:162	1.897	0.1684
rs1333049	C	0. 4310	0.444	0.499	1.1034 (0.8523–1.4283)	246:254, 200:228	0.564	0.4526

*p*-HW, *p* value for Hardy–Weinberg equilibrium analysis; ObsHET: Observed heterozygosity; PredHET: Expected or predicted heterozygosity. * Significant at *p* < 0.05; ** Significant at *p* < 0.0001. CI: Confidence interval.

**Table 3:** Significant haplotypes associated with CAD in Saudi Arabian population.

Block	Haplotype	Frequency	Case, Control Frequency	Chi Square	*p* Value
Block 1	AGTA *	0.732	0.701, 0.768	5.245	0.0220
Block 2	TGG **	0.465	0.568, 0.345	46.38	9.74 × 10^−12^
Block 3	GGGCC *	0.321	0.288, 0.360	5.647	0.0175
Sig. SNPs	TAAG *	0.147	0.106, 0.195	14.473	1.00 × 10^−4^
	TGGA **	0.147	0.214, 0.067	39.769	2.86 × 10^−10^

* Protective haplotype; ** Risk haplotype. Sig. SNPs: Block of significant SNPs (Order of Significant SNPs: rs564398, rs4977574, rs2891168, rs1333042).

**Table 4:** Comparison between two multivariate models with and without CAD associated SNPs. AUC: area under curve.

Model	Variables	AUC (95% CI)	*p* Value	Difference in ACUs (95%CI): Model 2–Model 1	*p* Value
Clinical variables only (Model 1)	Age, Gender, BMI	0.79 (0.73–0.84)	1× 10^−10^	0.08 (0.04–0.12)	0.000236
Clinical variables + SNPs (Model 2)	Age, Gender, BMI, rs1333042, rs2891168	0.87 (0.82–0.90)	1× 10^−10^		
